# Comparison of the Sensititre YeastOne antifungal method with the CLSI M27-A3 reference method to determine the activity of antifungal agents against clinical isolates of Candida spp.

**DOI:** 10.3906/sag-1909-97

**Published:** 2020-12-17

**Authors:** Rabiye ALTINBAŞ, Ayşe BARIŞ, Sümeyye ŞEN, Recep ÖZTÜRK, Nuri KİRAZ

**Affiliations:** 1 Department of Microbiology, Division of Mycology, Eskişehir City Hospital, Eskişehir Turkey; 2 Department of Microbiology, Division of Mycology Şişli Hamidiye Etfal Training and Research Hospital, İstanbul Turkey; 3 Department of Microbiology Zonguldak Public Health, Zonguldak Turkey; 4 Department of Infectious Diseases and Clinical Microbiology, Medical School, İstanbul Medipol University, İstanbul Turkey; 5 Department of Microbiology, Medical School, Namık Kemal University, Tekirdağ Turkey

**Keywords:** Candida spp., antifungal susceptibility, broth microdilution, sensititre yeastone, susceptibility test comparison

## Abstract

**Background and aim:**

Infections caused by Candida species are significantly increasing today, and invasive Candida infections are generally associated with high mortality. Early diagnosis and identification of Candida spp. is important for the determination of antifungal agents that will be used for treatment. The aim of the present study was to provide a better regimen for Candida infections in the future.

**Materials and methods:**

The Sensititre YeastOne (SYO) method was compared with The Clinical Laboratory Standards Institute (CLSI) reference broth microdilution (BMD) testing method. Endpoints of minimal inhibitory concentrations (MICs) were determined for both methods.

**Results:**

By using both methods, MIC values of micafungin, caspofungin, voriconazole, and fluconazole were lower than amphotericin B. The values obtained with the SYO method were in high categorical agreement for ecinocandins and amphotericin B. The results of voriconazole and fluconazole were in low categorical agreement. The categorical agreement between the SYO and the BMD results at 24 h was 82.1% for VORI and 98.4% for AMB. Values obtained with SYO method for all antifungal agents were in high essential agreement with the data of the CLSI reference BMD method. The essential agreement between the SYO and the BMD results at 24 h was 94.0% for MFG and 99.0% for AMB.

**Conclusions:**

The SYO method was ready-to use, so it appeared to be easier and more efficient for Candida isolates.

## 1. Introduction

The *Candida* species are opportunistic pathogenic organisms, but they may also develop superficial and systemic infections in the presence of predisposing factors. The presence of a central venous catheter, use of broad-spectrum antibiotics, prolonged stay in intensive care units, mechanical ventilation, parenteral nutrition, dialysis, immunodeficiency, and diabetes mellitus compose the predisposing factors for candidiasis [1].

Infections caused by *Candida* species are significantly increasing today. *Candida albicans* ( *C.albicans* ) is the most common species, but the burden of nonalbicans *Candida* species is increasing [2]. Nonalbicans *Candida* species are also known to have decreasing susceptibility to antifungal agents. Since the antifungal susceptibility pattern among *Candida spp.* may differ, rapid diagnosis and identification of *Candida spp.* is important for the determination of antifungal agents that will be used for treatment. Antifungal susceptibility tests provide useful information to clinicians in determining effective antifungal treatments [3]. As a result of early diagnosis and efficient treatment, the rate of mortality and resistant strains are both reduced [4]. The Clinical and Laboratory Standards Institute (CLSI) has developed a document for the antifungal susceptibility tests of yeasts. CLSI recommends the broth microdilution method (BMD) M27-A3, which is used worldwide in laboratories for testing the *Candida* species [5,6]. This method is complex and requires an expert and laborious testing process to be used as a routine method adapted to hospital laboratories. Therefore, there is a need for alternative test methods. Sensititre YeastOne (SYO) (Trek Diagnostic Systems, Cleveland, OH, USA) is a commercially-prepared broth microdilution panel with colorimetric growth indicator Alamar Blue that produces minimal inhibitory concentration (MIC) data for *Candida spp.* [7]. SYO is an excellent, easy to handle, and practical alternative method for antifungal susceptibility testing and is commonly used all over the world.

The aim of the present study was to compare the performance of the SYO microdilution assay with that of the reference CLSI M27-A3 BMD method in antifungal susceptibility testing of 129 *Candida* isolates.

## 2. Materials and methods

### 2.1. Candida isolates

A total of 129 *Candida* isolates were tested. The majority of isolates (n = 90) were obtained from blood cultures and the remainder from urine cultures (n: 29) and deep-site specimens (n = 10). Identification of each isolate was performed using conventional methods (germ tube formation, microscopic morphology in corn meal-Tween 80 agar (CMA) (HiMedia, India) and biochemical analysis API 20C AUX (bioMerieux, France) [8]. All specimens were inoculated on to the Sabouraud Dextrose Agar (SDA) (Sigma-Aldrich, Madrid, Spain) and incubated at 35 °C for 24 h. A germ tube test was performed for classification of *Candida* *albicans* and nonalbicans *Candida* . Positive germ tubes were further incubated at 45 °C to look for the growth. The strains from SDA were inoculated on CMA for morphological examination of the production of chlamydospores, blastospores, true hyphae, and pseudohyphae. They were also inoculated on to Chrom Agar Candida (HiMedia, India) from SDA; identification was made by color and morphology of the colonies according to the manufacturer’s instructions [9]. Repeated isolates from the same patient were excluded.

### 2.2. Antifungal agent and susceptibility testing

Antifungal susceptibility testing methods were validated using quality control strains of *C. parapsilosis* ATCC 22019 and *C.krusei* ATCC 6258 [5].

### 2.3. Inoculum preparation

Before the tests were performed, each isolate was subcultured to SDA to ensure its purity and viability. After 24-h incubation, standard 0.5 McFarland fungal suspensions were prepared with sterile 0.85% saline. The turbidity of each yeast suspension was adjusted with Trek’s nephelometer for SYO, and the reference method was performed simultaneously.

### 2.4. CLSI broth microdilution method

Broth microdilution testing was performed according to the CLSI M27-A3 reference method. Microdilution plates were prepared according to reference method for FLU, VRC, AMB, CAS, and MFG.

The CLSI BMD plates were stored at −70 °C until the analysis day. MIC values for all agents were read following 24 h of incubation. Endpoints for azoles and echinocandins were defined as the lowest concentration of drug that resulted in a prominent reduction (approximately 50% inhibition) of visual growth compared with the drug-free growth control wells. The endpoint of AMB was defined as the lowest concentration of the drug, which resulted in total inhibition (100%) of noticeable growth. MIC values for all agents were evaluated following 24 h of incubation according to the CLSI document [5]. Species-specific clinical breakpoints (CBPs) for MFG, CAS, VRC, and FLC were evaluated according to the CLSI M27-S4 document [5,6]. The epidemiological cutoff value (ECV) was used for AMB, an isolate showing a minimum inhibitory concentration (MIC) of ≤1.0 μg mL−1, considered as susceptible and those with >1 μg mL−1 as resistant [10]. ECV was used for *C. lusitaniae* and *C. kefyr* to categorize the isolates as S (wild-type) and R (nonwild-type); for *C. lusitaniae* , CAS (≤1/≥1 μg/mL), MFG (≤0.5/≥0.5 μg/mL), VRC (≤0.03/≥0.03 μg/mL), FLC and AMB (≤2/≥2 μg/mL), and for *C. kefyr* , CAS (≤0.03/≥0.03 μg/mL), MFG (≤0.12/≥0.12 μg/mL), VRC (≤0.005/≥0.005 μg/mL), FLC (≤1/≥1 μg/mL). ECV was used for *C. glabrata* , VRC (≤0.5/≥0.5 μg/mL) to categorize the isolates as S (wild-type) and R (nonwild-type) [11].

### 2.5. Sensititre antifungal susceptibility

SYO plates were shipped in sealed packages and stored at room temperature until testing was performed. The SYO panel trays contained serial two-fold dilutions of MFG, CAS, and VRC (0.008 to 8 μg/mL), FLC (0.12 to 256 μg/mL), and AMB (0.12 to 8 μg/mL). SYO panels were provided by Trek Diagnostic Systems. Stock inoculum suspensions of the *Candida spp.* were obtained from 24-h cultures on SDA at 35 °C. Susceptibility testing, reading, and interpretations of the results were performed in accordance with the manufacturer’s instructions. The dried SYO panels were rehydrated with the yeast suspension using an appropriate multichannel pipetting device by dispensing 100 μL into each well. Panels were covered with adhesive seals and incubated at 35 °C for 24 h in a non-CO2 incubator. MICs endpoints were read after 24 h of incubation. Evident yeast growth was observed as the color changed from blue (negative, indicating no growth) to red (positive, indicating growth) [7]. Susceptibility to MFG, CAS, VRC, FLC, and AMB was evaluated by using colorimetric microdilution panels. MIC values were evaluated using the CLSI M27-S4 document for MFG, CAS, VRC, and FLC; ECV was used for AMB.

### 2.6. Categorical agreement and essential agreement

To determine categorical agreement (CA) between SYO and BMD, a MIC result was required; major errors were classified as results of resistance to SYO and susceptibility to BMD. Very major errors were classified as results of susceptibility to SYO and resistance to BMD. Minor errors occurred when the result of one of the tests was susceptible or resistant and that of the other test was susceptible-dose dependent [10].

Essential agreement (EA) was defined in terms of discrepancies in MIC results of no more than +/– 2-fold dilutions between SYO and BMD. Results obtained by the BMD and by the SYO methods were calculated to determine the percentages of EA between MIC values [12].

## 3. Results

In this study, we compared the in vitro activities of 5 antifungal agents in 3 different groups (echinocandin, polyene, and azole) against different *Candida* isolates by the SYO method and BMD method, which is recommended by the CLSI. A total of 129 *Candida* isolates were defined, and the species distribution of the isolates were as follows: *C. albicans* (n = 42, 33%), *C. glabrata* (n = 15, 12%), *C. parapsilosis* (n = 37, 29%), *C. tropicalis* (n = 19, 15%) *,* *C. krusei* (n = 5, 4%), *C. kefyr* (n = 5, 4%), and *C. lusitaniae* (n = 6, 5%). Antifungal MIC range, MIC50, and MIC90 values using both SYO and CLSI BMD methods are shown in Table 1.

**Table 1:** In vitro susceptibilities of Candida spp. as determined by the Sensititre YeastOne and CLSI reference methods.

Species (%)	Antifungal drug	BMD method	MIC range (µg/mL)	MIC50	MIC90	GM
Candida albicans (33%)	Micafungin	SYO	0.008–1	0.008	0.06	0.008
		CLSI	0.06–1	0.06	0.125	0.06
	Caspofungin	SYO	0.03–4	0.06	0.25	0.03
		CLSI	0.06–0.06	0.06	0.06	0.06
	Voriconazole	SYO	0.008–8	0.008	0.25	0.008
		CLSI	0.06–16	0.06	0.06	0.06
	Fluconazole	SYO	0.5–4	0.25	2	0.12
		CLSI	0.125–2	0.125	0.25	0.125
	Amphotericin B	SYO	0.12–4	0.5	1	0.25
		CLSI	0,25–4	0.5	2	0.25
Candida glabrata (12%)	Micafungin	SYO	0.008–12	0.015	0.015	0.015
		CLSI	0.06–0.25	0.06	0.125	0.06
	Caspofungin	SYO	0.03–0.5	0.12	0.25	0.06
		CLSI	0.06–0.25	0.06	0.125	0.06
	Voriconazole	SYO	0.12–8	0.25	2	0.25
		CLSI	0.06–32	0.06	1	0.06
	Fluconazole	SYO	0.125–32	4	16	0.5
		CLSI	0.125–32	0.5	8	0.25
	Amphotericin B	SYO	0.12–4	0.5	2	0.25
		CLSI	0.5–2	2	2	1
Candida parapsilosis (29%)	Micafungin	SYO	0.05–4	1	2	0.25
		CLSI	0.06–8	0.125	2	0.06
	Caspofungin	SYO	0.03–4	0.5	1	0.12
		CLSI	0.06–4	0.06	0.5	0.06
	Voriconazole	SYO	0.008–16	0.06	2	0.008
		CLSI	0.06–8	0.06	1	0.06
	Fluconazole	SYO	0.12–16	1	8	0.25
		CLSI	0.125–8	0.5	2	0.25
	Amphotericin B	SYO	0.12–2	0.25	1	0.12
		CLSI	0.25–2	1	2	0.25
Candida tropicalis (15%)	Micafungin	SYO	0.008–1	0.03	0.06	0.015
		CLSI	0.06–4	0.125	0.5	0.06
	Caspofungin	SYO	0.015–4	0.12	0.25	0.03
		CLSI	0.06–1	0.06	0.06	0.06
	Voriconazole	SYO	0.008–8	0.25	8	0.12
		CLSI	0.06–32	0.06	16	0.06
	Fluconazole	SYO	0.12–4	2	4	0.5
		CLSI	0.125–16	0.25	2	0.125
	Amphotericin B	SYO	0.25–4	1	4	0.25
		CLSI	0.5–4	2	4	0.5
Candida kefyr (4%)	Micafungin	SYO	0.008–0.12	0.06	0.12	0.06
		CLSI	0.06–0.5	0.06	0.5	0.06
	Caspofungin	SYO	0.03–0.12	0.06	0.12	0.06
		CLSI	0.06–0.06	0.06	0.06	0.06
	Voriconazole	SYO	0.008–0.25	0.06	0.25	0.06
		CLSI	0.06–0.125	0.06	0.125	0.06
	Fluconazole	SYO	0.5–2	0.5	2	0.5
		CLSI	0.125–4	0.125	4	0.125
	Amphotericin B	SYO	0.5–1	0.5	1	0.5
		CLSI	0.125–4	1	4	1
Candida krusei (4%)	Micafungin	SYO	0.015–0.12	0.12	0.25	0.12
		CLSI	0.06–1	0.25	1	0.25
	Caspofungin	SYO	0.03–0.5	0.25	0.5	0.25
		CLSI	0.06–0.125	0.06	0.125	0.06
	Voriconazole	SYO	0.03–1	1	1	1
		CLSI	0.06–4	0.25	4	0.25
	Fluconazole	SYO	1–32	8	32	32
		CLSI	0.5–32	8	32	8
	Amphotericin B	SYO	0.25–4	0.25	4	2
		CLSI	0.125–4	0.5	4	0.5
Candida lusitaniae (5%)	Micafungin	SYO	0.015–0.06	0.06	0.06	0.06
		CLSI	0.06–0.25	0.06	0.25	0.06
	Caspofungin	SYO	0.03–1	0.12	1	0.12
		CLSI	0.06–0.25	0.06	0.25	0.06
	Voriconazole	SYO	0.008–0.06	0.03	0.06	0.03
		CLSI	0.06–0.125	0.06	0.125	0.06
	Fluconazole	SYO	0.25–8	1	8	1
		CLSI	0.125–32	0.25	32	0.25
	Amphotericin B	SYO	0.12–0.5	0.25	0.5	0.25
		CLSI	0.125–2	0.25	2	0.25

BMD: Broth microdilution; CLSI: Clinical and Laboratory Standards Institute; SYO: Sensititre YeastOne; MIC: Minimal Inhibitory Concentration

The essential agreement of each antifungal agent, according to the reference test, was examined at the species level. All antifungals showed strong essential agreement (> 90%) for all clinical *Candida spp.* isolates except FLC and AMB (87% and 87% agreement) for *Candida glabrata* and MFG (83% agreement) for *C. lusitaniae.* *Candida albicans* , *Candida parapsilosis* , and *Candida tropicalis* composed 75% of the isolates in our study, and the compatibility of these 3 species was found to be over 90% with all antifungals (AMB, FLC, VRC, CAS, and MFG). All *Candida kefyr* and *Candida krusei* were found to be 100% compatible with all antifungals.

CA was excellent for AMB (98.4%), for CAS (94.5%), and for MFG (90.7%); and good for FLC (83.9%) and VRC (82.1%). CA values between the SYO and CLSI method results was 92.2% (119/129) for MFG with 2 very major errors and 8 minor errors; 94.6% (122/129) for CAS, with one VME, 2 major errors and 4 minor errors; 82.2% (106/129) for VRC with 6 very major errors, 5 major errors, and 12 minor errors; 84.7% (105/124) for FLC with 5 major errors, 15 minor errors; and 97.6% (121/124) for AMB, with 2 very major errors and 1 major error. High quantities of very major errors were primarily found for *C.lusitaniae* , *C.krusei* , and *C.kefyr* . *C.lusitaniae* included 5 very major errors (83.3%) for VRC. *C.krusei* included 2 very major errors (40%) for micafungin, and *C.kefyr* included 2 very major errors (40%) for MFG, 1 very major error (20%) for CAS, and 1 very major error (20%) for VRC (Table 2).

**Table 2:** Category and percent agreement between SYO and CLSI reference broth microdilution MIC points.

Species(no. of isolates tested)	Antifungal agent	% of MICs by category			% error			CA%	EA(%)
		S	I/S–DD	R	VME	ME	Minor		
C. albicans (42)	MFG	95.2	2.4	2.4	0.0	0.0	2.4	97.6	95.0
	CAS	100.0	0.0	0.0	0.0	2.4	2.4	95.2	95.0
	VRC	97.6	0.0	2.4	0.0	0.0	9.5	90.5	100.0
	FLC	100.0	0.0	0.0	0.0	0.0	4.8	95.2	98.0
	AmB	92.9	0.0	7.1	4.8	0.0	0.0	95.2	100.0
C.glabrata (15)	MFG	80.1	13.3	6.6	0.0	0.0	20.0	80.0	93.0
	CAS	93.4	6.6	0.0	0.0	0.0	13.3	86.7	100.0
	VRC	86.7	0.0	13.3	0.0	6.7	0.0	93.3	100.0
	FLC	0.0	100.0	0.0	0.0	0.0	0.0	100.0	87.0
	AMB	100.0	0.0	0.0	0.0	6.7	0.0	93.3	87.0
C.parapsilosis (37)	MFG	91.9	5.4	2.7	0.0	0.0	8.1	91.9	92.0
	CAS	97.3	2.7	0.0	0.0	0.0	0.0	100.0	92.0
	VRC	78.4	8.1	13.3	0.0	0.0	5.4	94.6	100.0
	FLC	89.2	8.1	2.7	0.0	10.8	21.6	67.6	100.0
	AMB	100.0	0.0	0.0	0.0	0.0	0.0	100.0	100.0
C.tropicalis (19)	MFG	84.3	10.5	5.2	0.0	0.0	5.2	94.8	95.0
	CAS	94.8	0.0	5.2	0.0	0.0	5.2	94.8	95.0
	VRC	84.2	0.0	15.8	0.0	10.5	31.5	58.0	90.0
	FLC	89.5	0.0	10.5	0.0	0.0	26.3	73.7	90.0
	AmB	84.2	0.0	15.8	0.0	0.0	0.0	100.0	100.0
C.kefyr (5)	MFG	60.0	0.0	40.0	40.0	0.0	0.0	60.0	100.0
	CAS	0.0	0.0	100.0	20.0	0.0	0.0	80.0	100.0
	VRC	0.0	0.0	100.0	20.0	0.0	0.0	80.0	100.0
	FLC	80.0	0.0	20.0	0.0	0.0	0.0	100.0	100.0
	AMB	–	–	–	–	–	–	–	100.0
C.krusei (5)	MFG	60.0	–	40.0	40.0	0.0	0.0	60.0	100.0
	CAS	100.0	–	0.0	0.0	20.0	0.0	80.0	100.0
	VRC	80.0	–	20.0	0.0	40.0	0.0	60.0	100.0
	AMB	80.0	–	20.0	0.0	0.0	0.0	100.0	100.0
C.lusitaniae (6)	MFG	100.0	0.0	0.0	0.0	0.0	0.0	100.0	83.0
	CAS	100.0	0.0	0.0	0.0	0.0	0.0	100.0	100.0
	VRC	0.0	0.0	100	83.3	0.0	0.0	16.7	100.0
	FLC	83.4	0.0	16.6	0.0	16.6	0.0	83.4	100.0
	AMB	100.0	0.0	0.0	0.0	0.0	0.0	100.0	100.0
All Candida spp.	MFG	88.4	5.4	6.2	3.1	0.0	6.2	90.7	94.0
	CAS	93.8	1.6	4.6	0.8	1.6	3.1	94.5	95.0
	VRC	79.9	2.3	17.8	4.7	3.9	9.3	82.1	98.0
	FLC	81.5	14.3	4.0	0.0	4.0	12.1	83.9	96.0
	AMB	94.4	0.0	5.6	0.8	0.8	0.0	98.4	99.0

## 4. Discussion

Candidiasis is an important cause of morbidity and mortality in patients; thus, early diagnosis and therapy is important for preventing invasive candidiasis [13–15]. Antifungal treatment is often empirically started in patients with critical illness and continued after evidence of clinical improvement, even in the absence of positive mycological data. Inappropriate use of antifungal agents is associated with high costs, toxicities, and drug to drug interactions [16,17].

Antifungal susceptibility testing will play an important role while selecting appropriate medications. The main purpose of these tests is to enable practitioners to obtain clinical success during the treatment of fungal infections [18].

The CLSI BMD M27-A3 is the standard technique for susceptibility testing in microbiology laboratories, and in vitro results of the MIC determinations have been shown to correlate quite well with clinical outcomes; however, this test is complex and expensive. CLSI BMD requires many steps, including preparation of the drugs and solutions and manual inoculation, moreover, reading MICs is not easy. Also, the test results are affected by the concentration of the inoculum, the composition and pH of the medium, and the temperature and time of incubation [19–21]. Alternative methods have to be used because of the obvious reasons mentioned above [22]. One alternative method, SYO, is more easily applied, and no complex handling is required. In addition, this method has the advantage of facilitating the determination of endpoints [23,24]. SYO is an adapted susceptibility test method of the CLSI BMD method based on the M27-A3 standard for yeasts, which uses Alamar Blue as a colorimetric indicator. SYO is such an easy commercial system that it only requires adding a medium containing fungal inoculums [25–27]. Another advantage of the SYO method is that it allows an easy interpretation of the results through Alamar Blue. The SYO test method also provides standardization of antifungal tests in all countries. Furthermore, the SYO test method is suitable for poor laboratory conditions and slow laboratory turnaround times.

The SYO method has shown excellent results and could be an alternative in clinical laboratories. The CA results were similar with those found in Bertout et al.’s [28] results for FLC (83.9%) and VRC (82.1%).

A high rate of discrepancy was observed between the SYO and CLSI methods for VRC, and this occurred with *C.lusitaniae, C.tropicalis,* and *C.krusei,* (16.7%, 58%, and 60%, respectively). The discrepancy mentioned above may be due to the low number of strains, so it will be appropriate to repeat the test with a greater number of strains.

In the previous literature, Siqueira et al. [29] reported these values as 56.25% for both VRC and FLC, which can be considered low. Also, for *C. parapsilosis* , the SYO method exhibited a low performance only with FLC (67.6%). For *C. albicans, C. parapsilosis* , and *C. tropicalis* , the SYO method showed good performance with echinocandins, MFG, and CAS (CA > 90.0%). Bertout et al. [28] observed CA values of 72.6% and 94.1% while comparing SYO and BDM. In our study, these values were 82.1% and 98.4%. Siqueira et al. observed low CA for *C. glabrata* with CAS (68.75%). In contrast, *C. glabrata* for CAS showed good CA in our study (86.7%).

Pfaller et al. [30,31] observed an excellent CA for CAS and MFG (93.6% and 99.6%, respectively). Similarly, in our study, these values were 94.5% and 90.7%, respectively. Variations in the CA depending on the species and drugs tested were noted, and CA was found to be 16.7% and 100% in our study. Cuenca-Estrella et al. [12] tested susceptibility for *Candida spp.* with the SYO and BMD methods, as well. As a result, a high EA value (greater than 97%) was found with all antifungals tested. The EA value for AMB was 97.4%, 96.0% for FLC, and 95.5% for VRC. Similar findings were obtained in our study with EA values of 99.0%, 96%, and 98%, respectively, for AMB, FLC, and VRC. As in previous studies, the highest EA value was determined with AMB (99%) in our study.

Espinel-Ingroff et al. [32] found EA and CA values between the SYO and the reference BMD methods as 100% for echinocandins, MFG, and CAS in their study. We also found compatible results. Bertout et al. declared the EA rates between the SYO colorimetric method and the CLSI BMD method for FLC, VRC, AMB, and CAS as 70.6%, 80.4%, 92.2%, and 88.2%, respectively. These values were lower when compared with our values. On the other hand, they presented the CA rates as 87.3%, 86.3%, 72.6%, and 97.1% for FLC, VRC, AMB, and CAS, respectively. These values point to similar rates with our values except for AMB, which had a higher rate in our study. These researchers determined VME rate to be 0.9% (AMB, CAS) and 7.8% (VRC), and reported the ME rate as 2.9% (VRC) and 26.5% (AMB). They indicated that the lowest CA was for AMB (72.6%) [28].

Despite the limited isolates tested, we concluded that the SYO method has a good performance, and it is reliable for antifungal susceptibility testing. Nevertheless, VRC and FLC activity against the *Candida* species should be interpreted carefully when using SYO because we observed a low CA value (lower than 90%).

In this study, the SYO method showed excellent results for the most common *Candida* species ( *C. albicans, C.parapsilosis, C. glabrata* , and *C. tropicalis* ) with the exception of *C. glabrata* with MFG and CAS (80.0% and 86.7%, respectively), *C. tropicalis* with VRC and FLC (58,0% and 73.7%, respectively), and *C. parapsilosis* with FLC (67.6%). As a result, the SYO method could be an alternative method for antifungal susceptibility testing in clinical laboratories as recommended in Espinel’s study [32].

This study is the first research study comparing differences between the SYO method and the CLSI reference BMD method for testing the in vitro susceptibility test of over 100 *Candida* strains in Turkey. Strictly speaking, our study contained susceptible strains instead of resistant ones. Therefore, it is recommended that more research should be carried out with a large number of strains, including resistant *Candida* isolates. The SYO method was in excellent correlation with the reference method CLSI BMD for all antifungal drugs except VRC and FLC in our study, so it can be inferred that the SYO method is less suitable for susceptibility testing of VRC and FLC.

## 5. Conclusion

Since the SYO method is simple, easy to apply, and compatible with the reference method, it can be used instead of the CLSI reference BMD method while testing the antifungal susceptibility of the *Candida* species. Our study confirmed that the SYO method was an efficient and effective alternative to the reference method in determining the susceptibility of *Candida* isolates in clinical laboratories.

## Informed consent

No ethical approval was required as the research in this article was related to microorganisms.
